# Risk Governance in the Early Pandemic: Governance Roles and Coleman’s Taxonomy of Social Actors

**DOI:** 10.1177/00027642221132175

**Published:** 2022-11-05

**Authors:** Jeremy Schulz

**Affiliations:** 1 Institute for the Study of Societal Issues, University of California, Berkeley, CA, USA

**Keywords:** sociological theory, James Coleman, natural persons, risk governance, COVID-19

## Abstract

This article takes the ongoing conversation around risk governance in the context of the early stages of the COVID pandemic in a new direction. It does so by connecting public health risk governance to James Coleman’s formulation of social actorhood in the contemporary US. Risk governance across a variety of social settings can be fruitfully conceptualized according to Coleman’s taxonomy of natural and constructed social actors. Unveiling the risk governance schemes operating within distinct social settings is a matter of teasing out the governance roles played by the three primary types of social actor introduced by Coleman: natural persons, agents/principals, and citizen-sovereigns. The parts played by these types of actors are examined within distinct meso-level settings such as households, employment settings, public-facing retail settings, and colleges. In this way, the study is able to distinguish specific governance schemes in terms of how they mobilize particular kinds of social actors characterized according to Coleman’s taxonomy. The study represents a step toward developing an account of risk governance which can accommodate a wide variety of actors, settings, and dynamics within a coherent theoretical framework. In carrying out this exercise, this study applies sociological theories to open a window into crucial aspects of risk governance during the pandemic era.

If risky encounters are the basis for much of the health risk associated with the pandemic, the governance of risky encounters assumes enormous importance for both individuals and societies contending with this omnipresent threat. The ubiquity and visibility of risky encounters in pandemic society and the correlative ubiquity and visibility of risk governance have created an opening for novel theoretical approaches to risk governance which address the distinctiveness of particular categories of social actors at different levels of social organization (Smelser, 1995). For instance, one recent contribution to the work around risk governance stresses the divide between different ethics of risk and harm mitigation from the standpoint of the individual. In the context of regulating food allergies in shared social spaces—namely passenger planes in the United States—diametrically opposed perceptions of risk governance come into play (DeSoucey & Waggoner, 2022). In this setting, some individuals see the mitigation of risk around food allergies on planes through the lens of a hyper-individualistic understanding of personal responsibility, while others view personal responsibility through a decidedly relational and collectivistic lens. Questions around risk management in this context turn on whether individuals are responsible for the health and safety of others with whom they come into contact as well as their own health and safety.

While undoubtedly valuable, the risk society and risk culture perspectives divert the theoretical gaze away from key insights into the ways that risk governance works in particular social domains and vis-á-vis particular social actors. In order to redirect this gaze, this article revisits James Coleman’s oft-neglected theoretical framework for characterizing what Meyer and Jepperson call social “actorhood” (Meyer & Jepperson, 2000). Coleman’s framework lends itself to conceptualizing the governance of risky encounters in ways that recognize the specificity of lower-level and higher-level social actors, domains, and relations. By examining risk governance in the pandemic through this theoretical lens, we can see how risk governance measures reflect the allocation of rights and agency among distinct social actors, distinct levels of social organization, and distinct kinds of social relations. At the same time, it throws into sharp relief the way in which the mitigation and governance measures themselves realign the distribution of agency—conceived as rights of action—across these various domains and levels.

The character of the pandemic has made it clear how health risk is shaped by the ubiquity of interaction and social encounters. From an epidemiological standpoint, a large part of the overall disease risk stems from social encounters as opposed to the handling of inanimate objects or the breathing of contaminated air. Much of the health risk in pandemic society derives from risky encounters between infected and uninfected individuals. Moreover, although some individuals stand a greater chance of infection and illness than others, everyone can transmit the virus and thereby serve as a disease vector (Will, 2020). Even now, during the post-variant/post-vaccination phase of the pandemic, medical studies have conclusively established that both unvaccinated and vaccinated people can transmit the virus to other vaccinated people, meaning that risky encounters are ubiquitous, even in a nearly totally vaccinated society.

Sociologically, risky encounters are themselves unevenly distributed across different parts of society along a variety of sociodemographic axes and socioeconomic axes. For instance, workers in “interpersonal” industries and fixed-located industries are most exposed to risky encounters, as contrasted with those who can work remotely and away from other people, signaling a growing risk divide between more and less favorably situated workers (Fassin & Fourcade, 2022, Grusky et al., 2021, Holst, 2021). Moreover, at the population or community level, the volume of risky encounters itself proves decisive for viral transmission. On average, dampening viral transmission in more encounter-intensive communities—as contrasted with communities characterized by a lower density of social encounters—requires more intensive mitigation measures aimed at individuals (Moody et al., 2021).

Furthermore, exposure risk is transmitted through complex webs of risky encounters in potentially long chains cutting across distinctive social settings.^1^ A single risky encounter involving one set of social actors in one arena can create the conditions for other risky encounters downstream from the focal encounter in a distinct social context. Exposure risk originating in risky encounters taking place in private settings among one set of individuals can easily propagate into exposure risk for other individuals in public settings and vice versa. Because exposure risk flows across social settings, to the extent that individuals transition in and out of environments on a regular basis, they are effectively importing and exporting exposure risk from one risky encounter to another and from one environment to another. For instance, upstream risk originating in risky workplace encounters can flow to households and vice versa. Indeed, when contagious individuals take part in risky encounters unfolding in employment environments—for example, workplaces—they may create the conditions for downstream risky encounters in their own households as well as others’ households.

## Risk Governance Schemes

While much recent literature on risk and risk governance emphasizes that in contemporary societies the individual is positioned as the sole responsible party where risks to his or her health are concerned (DeSoucey & Waggoner, 2022, Shamir, 2008), risk governance does not begin and end with the individual. Just as exposure risk can propagate along chains of risky encounters located in different social environments, the governance of risky encounters through behavioral mitigation measures means that multiple levels of social organization (Smelser, 1995) and diverse types of social actors are implicated in the governance of risky encounters and exposure risk more generally. These mitigation measures can constitute diverse kinds of social actors as targets, including individuals, groups, formal organizations, and communities, diverse social entities within diverse social contexts. At the same time, the social actors responsible for implementing and enforcing these mitigation measures themselves span the full range of social actors (Renn, 2008 p.132). On some occasions, individuals are responsible for either initiating or implementing rules, while on other occasions the task of rule initiation/implementation may fall to organizations, communities, or governments or some combination of these actors. In other words, governance measures must recognize that social actors can play one or more of the three basic governance roles implicit in all governance and mitigation measures, namely that of target, implementer, and initiator. Therefore, a theoretically well-grounded understanding of the governance of risky encounters in pandemic society entails a theoretically rigorous account of what kinds of social actors play the roles of enforcement target, implementer, and initiator. Such an account, in turn, necessitates revisiting both the familiar distinction between the public domain and the private realm and the less familiar theoretical framework developed by James Coleman, namely his model of social actorhood.

From the outset, it is clear that risky encounters can take place in either public or private settings. This implicit reference to the private/public distinction is common in both social science and journalistic accounts of risky encounters and risk mitigation. It has been observed on numerous occasions that the majority of risk mitigation measures aimed at risky encounters, termed “non-pharmaceutical interventions” in epidemiological parlance, are geared toward public settings instead of private settings (Brauner et al., 2021). Public-facing risk mitigation measures such as lockdowns, movement restrictions, confinement, and closures of businesses are designed to effectively reduce the volume of risky encounters among strangers, without necessarily restricting risky encounters within private settings or the “intimate” sphere (Basuare et al., 2021, Kumar & Makarova, 2008). Such public-facing collective mitigation measures in effect reconfigure the ecology of risky encounters across the public/private divide, minimizing opportunities for risky encounters in public settings, while leaving private settings relatively unaffected.

But it is insufficient to grasp the governance of risky encounters and exposure risk as purely explicable in terms of the distinction between the public and private realms. Conceptualizing governance measures necessitates considering taxonomic differences among types of social actors and relations which are not captured by the public–private dichotomy. In fact, an adequate theoretical account of risk governance measures requires an engagement with the tier identities incorporated into the agency-rights framework proposed by James Coleman. Such an understanding of risk governance must accommodate the taxonomic distinctions between different tiers of social organization and types of social actors introduced in this theoretical framework.

Here we bring together the notion of governance roles with the conception of social actorhood introduced in Coleman’s agency-rights framework. Through the integration of these frameworks, we can better grasp the dynamics of different governance schemes which position different kinds of social actors as governance targets, implementers, and initiators or occupants of more than one of these roles. As we will see, in different governance schemes corresponding to different social settings, social actors of different types can assume one or more of these governance roles. Across all social domains, the governance and mitigation of exposure risk necessarily involves not only natural persons, but also persons with activated tier identities as agents (employees), principals (owners), as well as those with higher-tier background identities as “citizen-sovereigns” (Coleman, 1991). Contrast, for example, the governance schemes characteristic of settings populated exclusively by natural persons versus the schemes corresponding to settings with natural persons and corporate actors such as public-facing employment environments (e.g., retail stores). As we will see, there is a vast difference between the kind of risk governance scheme feasible in private natural person settings versus settings inhabited by constructed social actors, whether those with activated higher-tier identities or background higher-tier identities. The governance scheme which operates in the social settings populated by higher-tier social actors, targets, implementers, and initiators is linked together in complex chains of authority (Reed, 2020, 2017). In such a chain, authority can be delegated from one type of social actor to another type of social actor and some actors are positioned to implement protocols and measures on their own behalf, but also on behalf of other actors.

## Social Actorhood and Risk Governance

In Coleman’s framework of distinctive social tiers, the foundational level of social order is defined as “primordial” or “natural.” What is dubbed the “constructed” tier of social relations is grafted onto this primordial tier when individuals join or form intentional intangible corporate actors such as formal organizations or become embedded in spontaneous intangible corporate actors such as nation states and subnational political collectivities (Coleman, 1991, 1990, 1982). In Coleman’s agency-rights approach to social organization, the natural and constructed types of social actor engage in distinctive social relations and are equipped with distinct juridically recognized rights and responsibilities. It is important to note that particular tier identities can be activated or dormant both across particular social settings and within particular social settings, and that identities can be combined in multiple identity configurations (Coleman, 1990, pp. 542–548). Across a particular social group or community, a multiplicity of tier identities are activated and inactivated in the course of a given day.

The foundational tier identity for Coleman is the identity of the natural person, a legally recognized social actor which, under most circumstances, is associated with the embodied individual (Coleman, 1991, p. 6, Coleman, 1990, pp. 540–546, Fuchs, 2001, p. 10). This foundational tier identity, however, is not the only identity manifested in the course of everyday life in contemporary societies. In fact, it shares the stage with several higher-tier identities. These higher-tier identities come in two primary flavors: the two complementary activated higher-tier identities (agent/principal) and a background higher-tier identity (citizen-sovereign). Whereas the agent/principal pair of identities must be activated by social actors in “concrete interactions” which presuppose ties with other social actors (Coleman, 1990, p. 543, pp. 545–546), the background higher-tier identity of the citizen-sovereign does not have to be activated in this way.

In Coleman’s model, the activated higher-tier identity of agent coincides empirically with the familiar juridically defined category of employee. In Coleman’s framework, when individuals act in their capacity as legally empowered agents with respect to a principal—the owner of a corporate entity—they bear particular rights and responsibilities defined in relationship to the complementary rights and responsibilities of the principals who control the corporate entity. The activated identity of agent is therefore part of a triadic identity configuration which implicates the corporate level of social organization. In this triadic relation, the agent acts in the “name of” the ultimate authority or principal, according to the terms of the implicit or explicit employment arrangement (Reed, 2017; Coleman, 1990, pp. 166–175). Where individuals are acting as employees of a formal organization, for instance, they express an activated agent identity defined by their employment relationship to the organization’s owners or principals, a relationship whose termination releases them from this particular identity. The rights and responsibilities of the agent are registered and codified within the legal-juridical system. Participation in these kinds of triadic social relations also implies participation in authority relations wherein one actor delegates authority over a specific area of action to another person or juristically defined actor. This social actor is juristically designated as the agent. For working-age adults, the higher-tier identity is typically activated at work, whereas the identity of the natural person is activated in the private realm (Coleman, 1982, 1990, 1991, pp. 542–545). However, it is important to recognize, particularly in an era when remote work and work from home is more widespread than ever, that the agent identity can be activated in settings which would be typically classified as private. This heightened activation potential outside of the conventional workplace renders the activation of the agent identity a more or less continuous process. Such a process of activating and deactivating the agent identity may therefore be likened to the “switching” process observed by social psychologists studying the interface between work and personal life (Cornwell, 2013; Nippert-Eng, 1996; Schieman & Badaway, 2020).

However, in Coleman’s framework, there is another constructed identity—often overlooked—which is not activated and deactivated in this way, namely the identity of the “citizen-sovereign.” This higher-tier background identity differs in important ways from the activated tier identities evident in the employment setting. As a consequence, it has different ramifications for the governance of risky encounters. This higher-tier identity differs from the natural person identity, but also can be differentiated from the constructed identities associated with intangible yet intentional corporate bodies, namely formal organizations, as it is not mediated through any agent–principal relationship. The citizen-sovereign is defined by Coleman as the identity which natural persons assume by birth or naturalization once they are incorporated into a sovereign political collectivity or body with correlative rights and obligations, as well as the conjoint system of authority it embodies (Coleman, 1990). In their capacity as citizens of a particular nation-state or subnational political collectivity, citizen-sovereigns are also exercising rights and discharging responsibilities attached to their membership in a specified corporate body—except that in this case it is a “primordial” corporate body (the political collectivity) rather than a purpose-built economic organization composed of interchangeable positions (Coleman, 1990, p. 597). Those individuals juristically constituted as citizens (political principals) belong to the corporate body of the citizenry.

As citizens of national polities and subnational polities, citizen-sovereigns are members of the nation-state as well as particular subnational political collectivities, *sui generis* corporate actors which can exercise legal authority over private corporate actors as well as natural persons (Coleman, 1991, p. 32). As members of the corporate body of the nation-state and subnational-state(s), citizen-sovereigns can be considered as both the subjects and beneficiaries of the underlying social contract (Coleman, 1990, p. 327). In addition, the corporate “body” of the polity or political activity consists of natural persons who are its members rather than functional positions (Coleman, 1990, p. 597). Finally, the background identity of citizen-sovereign differs fundamentally from the activated identity of agent from the perspective of authority relations. The agent identity belongs to the “disjoint” authority structure, wherein rights of control over a particular sphere of action have been relinquished by the agent to the principal under defined conditions, typically in exchange for monetary payment (Coleman, 1990, p. 427). Membership in a “disjoint” system of authority represented by the authority structure of a corporate organization does not presume a coincidence between the interests of the employer and any of the employees (Coleman, 1990, p. 74). In the case of the citizen-sovereign identity, the presumption is that the interests of the citizen do in principle align with the interests of the political collectivity.

Where the governance of health risks is concerned, in Coleman’s framework, the government can exercise authority over citizen-sovereign individuals, provided that it is curbing health risks which stem from the actions of its own agents. This is clearly evident in the case of government-provided services, for example. If citizens of a particular county bear health risks because of the negligence of the county government—because of unclean water for example—the government bears the responsibility for mitigating these risks and can compel its own agents or employees to mitigate the risks for the sake of protecting its own citizenry (Coleman, 1982). According to Coleman’s framework, these lower-level governments and authorities are exercising authority delegated by the sovereign citizenry over matters of public health. Their authority derives from membership in the corporate body of the relevant political collectivity. Seen in this light, whereas the agents of the local government assume the role of implementers, the citizen-sovereign individuals become the targets of the governance measures, the implementers of the governance measures, and even the initiators of these measures, since the authority exercised by the government is *de jure* borrowed from the sovereign citizenry.

In the following sections of this paper, we will apply Coleman’s taxonomy of social actors together with the tripartite framework for understanding governance roles across different social settings. In this way, we can get a sense for how risk governance operates in these different settings. We will begin with settings where natural persons assume all three governance roles, settings such as private homes and other private spaces. We then move on to cases where the targets are natural persons in private settings, but where governmental authorities initiate and/or implement mitigation measures, creating an ambiguity around the relationship of government authority to natural persons. From there, we shift to an examination of risk governance in employment environments—for example, workplaces—where individuals acting as agents for other private parties such as formal organizations are positioned as implementers of governance measures vis-á-vis other agents. Further complexity arises in retail settings where natural persons (i.e., customers) and agents interact with one another, and where agents are often put in the uncomfortable position of implementing measures aimed at controlling customers’ behavior. This tour concludes with a brief overview of yet another context—the residential college—where students (“customers”) are contractually bound by their ties to the corporate entity to observe protocols which with they promise to comply, and where no one is put in a position of implementing measures vis-á-vis either other students or staff of the college.

## Natural Persons as Targets, Implementers, and Initiators

On one reading of Coleman’s actorhood framework, modern society consists in part of social environments populated exclusively by natural persons rather than constructed social actors. This type of foundational tier identity is characteristic of both private households and some kinds of public gatherings. In addition, in both private and public settings where the only social actors are natural persons, all three governance roles fall to natural persons. Indeed, the implementation of an informal risk agreement does not require the direct or indirect participation of corporate meso-level social actors such as formal organizations (Smelser, 1995) or the direct intervention of government agents through institutional domains such as the polity, the economic system, or the legal system (Abrutyn, 2014). Where natural persons serve as both targets and initiators/implementers of mitigation measures, the governance of risky encounters is a matter of informal decisions, habits, dispositions, and agreements rather than formalized rules and sanctions imposed by external institutional actors, whether private corporate actors or agents of the state (Coleman, 1982, p. 96). In these social formations, deliberation, negotiation, sanctions, and encouragement play key roles (Coleman, 1991, p. 14).

If we characterize the social environment of the private household as a social setting composed exclusively of natural persons, the governance of risky encounters within the household becomes a matter of facilitating compliance among interdependent natural persons who occupy one or more of the governance roles outlined previously, namely governance target, governance implementer, and governance initiator.

In strict natural person settings, therefore, optimal exposure risk governance presupposes that natural persons not only serve as targets of risk mitigation measures themselves, but also initiate these measures and implement them where necessary vis-à-vis other natural persons in their social environments. Natural persons may choose to comply with the governance and mitigation measures put forward by other natural persons in their social environment, such as kin or friends. Alternatively, they may comply with mitigation measures initiated and/or implemented by higher-tier social actors such as state agents and employers. Of course, both compliance and monitoring can only be achieved with high degrees of interpersonal trust as well as a recognition of interpersonal interdependence among the members of the social environment (Fine, 2010; Luhmann, 2017 [1973]). Governance breaks down where such trust and acknowledgment of interdependence is lacking, and household members are unable or unwilling to either comply with rules or monitor compliance among the other natural persons belonging to their household or other households. Indeed, with natural person settings such as kin groups, mutual monitoring and enforcement could easily fuel conflict and disharmony.

It is possible even for non-kin groups to collectively settle on informal risk governance approaches which promote compliance among group members and minimize the need for mutual monitoring. An illustrative example of such a group is the eleven-person “Manor of Being” (SF Chronicle, September 9, 2020), a small-scale co-housing community in San Francisco. In the early days of the pandemic, the group settled on an agreement whereby everyone adopted a quasi-formal process for budgeting or allocating the amount of risk for each member using an online risk allocation instrument (www.microcovid.org). They collectively availed themselves of a formalized protocol (Törnqvist, 2021) to clarify and control the riskiness of the encounters occurring outside the confines of the commune and translating into risk for commune members. In this way, the parameters for risky encounters inside and outside the commune were established collectively, through the adoption of a specific monthly risk budget which members could “spend” in various ways, with some allowances for personal proclivities. The implementation of the risk budgeting instrument was left to individual members, but the group as a whole was collectively responsible for enforcing the agreement. Here we see that the governance of exposure risk within a setting populated exclusively by natural persons requires the participation of individuals as both compliant targets and implementers vis-à-vis other members of the group.

## Natural Persons as Targets, Governments as Initiators

Where governance measures do not arise organically from the natural person tier of the social structure, but are imposed by governments, it becomes challenging to assign governance roles to particular kinds of social actors. For instance, some mandates apply directly to private settings where, under one interpretation, only natural persons interact. For example, the State of Pennsylvania Department of Public Health issued a directive in 2020 which mandated masking for any individuals mingling with others outside of their own household, even if they are doing so in private settings such as homes. More recently, Santa Cruz County health authorities have directed residents to wear masks when copresent with non-household members, even within private homes. The Santa Cruz health order stipulates that a violation of the order constitutes a misdemeanor “punishable by fine, imprisonment, or both” (County of Santa Cruz 2021). As for enforcement, the directive states that it is left in the hands of “enforcement officers”—namely agents of the government acting in their official capacity. Here it is unclear whether the targets of these governance measures are considered as strictly natural persons or also as citizen-sovereigns, since the state is not allowed to intrude directly into the kinds of settings where natural persons interact.

The ambiguity of this ordinance lies in the fact that it lends itself to at least two distinct interpretations in relation to Coleman’s taxonomy of social actors and relations. This ambiguity stems from the difficulty in identifying the targets and implementers of such risk mitigation measures as either natural persons or citizen-sovereigns, an ambiguity which Coleman notices in his own treatment of risk governance in light of tier identities (Coleman 1982, p. 86). In the first natural person interpretation, the private realm is constituted as a site where local residents *qua* natural persons are implementing mitigation measures which affect themselves and other natural persons—whether members of their own household or some other household. However, there is an alternative citizen-sovereign interpretation wherein private citizens complying with these guidelines and enforcing these guidelines in effect act as citizen-sovereigns rather than natural persons detached from all corporate bodies. On this reading, whether they serve as targets, implementers, or initiators of the mitigation measures, these individuals are acting in their capacity as citizen-sovereign members of the corporate body of the polity itself. On this reading of these directives, the targets and implementers of the directives are responsible for their own well-being as well as the well-being of their fellow citizen-sovereigns.

## Agent–Agent Settings: Employment Environments

While the governance of risky encounters in strict natural person environments entails the participation of natural persons in all three governance roles (target, implementer, and initiator), the governance of risky encounters becomes more complex when higher-tier social actors enter the scene. It is easy to identify social settings where such actors play important roles. Perhaps the two most salient environments are employment environments—where corporate principals and agents interact—and points of retail purchasing where agents and/or principals encounter retail customers in their capacity as natural persons. In employment environments, the responsibility for enforcing protocols governing risky encounters is distributed across different classes of social actor.

From the perspective of Coleman’s approach to social actorhood, employment environments are distinguished from natural persons settings such as households. Formal employment environments are inhabited by agents (e.g., employees) and principals attached to one or more corporate actors. In employment environments, employees and employers—defined as agents and principals—establish relationships structured indirectly by the state which takes on the role of institutionalizing third party (Luhmann, 2014 [1972]). Finally, employment and points of consumption are not equivalent to workplaces and retail sites where tier identities are concerned, as work and consumption can take place without the participation of higher-tier corporate social actors.^2^ Indeed, the distinction between employees and “non-employee workers” figures prominently in some COVID-19-related protocols (e.g., NYC https://www1.nyc.gov/site/doh/covid/covid-19-vaccine-workplace-requirement.page).

In employment environments, risky encounters can happen among agents attached to the same corporate actor or agents connected with different corporate actors, as well as owners/principals of their own organization or other organizations. The enforcement of risk mitigation protocols in this context implicates the owners or principals as well as the employees. In US jurisdictions, the principal’s general “duty of care” obligations toward employees are legally enforceable by both US federal and state governments (federal and state OSHA) and stipulate that the owner (i.e. principal) bears legal liability for violations of these rules even if the violations arise because of the conduct of her employees or agents (Coleman 1982, p. 102). However, in practice, legal liability for employers is not usually triggered unless the employer can be shown to be violating safe workplace regulations as they are set forth by government authorities. Indeed, while employing organizations have a legal obligation to minimize risky encounters for employees, in the US federal and state governments have afforded employers broad leeway in interpreting health and safety standard in regards to COVID exposure risk. While fines for serious OSHA violations are extremely rare, due to poor enforcement. In addition, fines for violations are capped at amounts which rarely pose financial risks to large enterprises.^3^ Thus, while in principle risky encounters among employees are treated as a special class of risky encounter in which health risk is translated into legal liability risk for the corporate principal identified with the employing organization, in practice the health risks to the employee far outweighs the liability/economic risks to the employer, particularly for employers with substantial economic resources. Thus, where workplaces are settings for potentially risky encounters among employees, owners typically face relatively little legal or economic risk.

In most US health jurisdictions, employers (principals) bear responsibility for ensuring that the workplace operated by the corporate body they own conforms to public health protocols. Failure to observe what legal authorities dub the “duty of care” can be considered culpable negligence on the part of the firm’s owners or principals (Hemel & Rodriguez, 2020), resulting in legal liability for them. Governance schemes which apply to employment environments also typically stipulate that employees have the obligation to inform employers of their health condition if they have reason to believe that they have contracted COVID. This is the case even in jurisdictions which have relatively “light” mitigation requirements.^4^ The employees are then supposed to monitor each other on behalf of the employer—their principal—and yet enforcement responsibility lies with the management of the business, and ultimately with principals of this corporate entity. In addition, employees must decide whether to report fellow employees or principals for violating risk mitigation protocols, simply warn them against violating these measures, or turn a blind eye to their violations. In practice, this means weighing loyalty to their peers, most likely fellow employees, against loyalty to the employer, the risk mitigation measures, and the overarching goal of public health itself (Pershing, 2003).

This type of governance scheme involves heterogenous types of social actors in multiple governance roles, giving rise to a complex governance chain involving employers (corporate principals) and employees (corporate agents) bound by codified and formalized rules and regulations (Stinchcombe, 2001). Both agents and principals serve as targets, monitors/implementers, and initiators of the governance measures. Whereas the targets of risk mitigation measures are classified as agents according to Coleman’s framework, the enforcement of these measures falls to both the owners or principals of the corporate entity as well as their agents.

## Agent-Natural Person Settings: Consumer-facing Retail Environments

A very different risk governance scheme materializes in environments where employees interact with customers, natural persons who by definition are exempted from any agent–principal relationship relevant to the organization. Recognized by Coleman as participants in a special class of “customer–store” (Coleman, 1990, p. 547) interaction, customers interacting with employees at points of consumption (defined legally as “public accommodations”)^5^ such as retail locations, restaurants, malls, mass transit facilities, and so forth are engaging in asymmetric encounters. In these settings, risky encounters can take the forms of asymmetric interactions between employees (agents) and customers (natural persons) in addition to symmetric encounters among employees. Points of consumption therefore differ from employment settings where only employees are present (e.g., warehouse facilities).

The governance scheme applicable to such points of consumption in the United States varies, depending on whether the environment is controlled by private parties versus governmental entities. Where government-controlled points of consumption are concerned, the risk governance scheme can differentiate between the mitigation of risky encounters among agents and risky encounters between agents and customers. For instance, in some of the objectively riskiest settings, such as air travel facilities regulated by the federal government, customers are exempted from vaccination requirements mandatory for employees.

Governance schemes which apply to privately owned and managed points of consumption and public accommodations vary among health jurisdictions. In some US health jurisdictions, privately owned retail businesses have the legal right to make mask-wearing or social distancing a condition of entry for customers, whether or not they are mandated by state or local health authorities (Hemel & Rodriguez, 2020, pp. 4–6). During the post-vaccination stage of the pandemic, some health jurisdictions required retail establishments and points of consumption to verify customers’ vaccination status. Furthermore, under these guidelines, the owners of the establishments were responsible for enforcing the vaccination requirements and non-compliant establishments could be reported anonymously to the public health authorities for violations.^6^ As of late 2021, the city of San Francisco had also decided that private businesses must demand verification of vaccination status from their customers.^7^

For instance, if the business requires proof of vaccination from customers, which is legally allowable in the United States, then it also falls to employees to monitor compliance with this requirement on the part of the business and its owner(s). Business owners and their agents are empowered to remove non-compliant customers from their establishments for violating this directive. Thus, here we have an example of a governance scheme where agents are monitoring natural persons on behalf of principals who are themselves carrying out directives issued by the local health authorities, constituting a complex chain of governance incorporating multiple types of social actors.^8^ At the same time, if employees fail to do so, and the business is reported to the authorities, then the legal liability for non-compliance falls on the owners. Indeed, in jurisdictions with relatively strict enforcement policies such as San Francisco, the enforcement of mitigation measures on the part of corporate agents has, in some cases, provoked strong resistance from either the employees or the owners of the business, as was evidenced in the case of the In-N-Out burger chain in California during the fall of 2021.

During the mandate stage of the pandemic, asymmetric encounters among agents and natural persons at public-facing retail venues served as the flashpoints for a variety of tensions and conflicts. During the pre-vaccination period of the pandemic, even when behavioral protocols such as mask-wearing were mandated by public health authorities in the United States for customers, local law enforcement personnel seldom intervened directly to ensure customer compliance or sanction violations. At the same time, to the extent that customers refused to follow the policies of the private business, they had to be policed by employees or store owners (e.g., principals) when other customers would not intervene on their behalf. This burden of policing risky encounters thus falls disproportionately on employees and owners of public-facing organizations rather than natural persons or agents of the state.

In many US health jurisdictions, employees of consumer-facing organizations face an even heavier monitoring and implementation burden during the post-vaccination phase of the pandemic. In San Francisco, for instance, as of November 10, 2021 “staff” of restaurants and other designated indoor customer-facing businesses are required to validate patrons’ proof of vaccination and cross-check this proof with a valid ID.^9^ Non-compliance with this rule can result in the closure of the establishment or the levying of a fine. Similar governance schemes can be identified even in health jurisdictions located in contrasting political landscapes where opposition to health mandates is widespread. For instance, in the summer of 2021 when COVID cases were spiking in Dallas, Texas, the county judge ordered that restaurants within the county compel employees and customers to wear masks while inside the establishment.^10^ Implementation, however, was left up to the restaurant owners and their employees, while customers could only be expelled for non-compliance. The same type of enforcement scheme in consumer-facing organizations appears when the protocols are initiated and applied by private corporate actors such as “big box” retailers.^11^ The character of this enforcement scheme is even evident in US states such as Florida, where state-level authorities tried to block private organizations from imposing vaccination requirements on customers by levying fines on organizations which do so.^12^ Applying to all customer-facing or public-facing organizations which interact with the public, the ban is directed at business owners—namely principals—rather than employees or agents, as employees cannot be sanctioned directly for setting up their own vaccination protocols independently of the business owner or head administrator.

These governance schemes single out employees—social actors with activated agent identities—as the implementers of risk governance measures which apply in customer-facing settings. Though these rules originate from the government and render the principals as the legally responsible parties, the implementers are nonetheless very likely to be the employees of the establishment and therefore classifiable as agents. Employees thus become implementers of mitigation measures vis-à-vis each other as well as those natural persons who are physically on the premises of the establishment. As agents of the establishment, they occupy dual governance roles in relation to multiple classes of social actors. The customers, on the other hand, are considered strictly as natural persons. As such, they may be defined as targets of the governance measures, but not as monitors or implementers of the measures vis-à-vis others.

## Contractually Bound Natural Persons: Students at Colleges

As we have seen, consumer-facing businesses provide the settings for governance schemes where agents and sometimes principals fulfill multiple governance roles of target and implementer vis-à-vis each other and the natural persons who are the establishment’s customers.

However, this is not the only kind of governance scheme operating in the so-called public settings. Whereas the retail setting is one in which employees occupy multiple governance roles with respect to multiple classes of social actors, other public settings may implement governance schemes in which natural persons are contractually bound to follow mitigation rules under a range of penalties. In these contexts, we observe a governance scheme rooted in explicit bilateral contracts between natural persons and the organization or corporate entity. Perhaps the clearest example of this type of setting is the American university campus.^13^ As an institution, the college campus is the stage for risky encounters among social actors with at least two distinct types of social actor, agents (employees), and natural persons (students). Unlike a consumer-facing business establishment, however, colleges can maximize compliance with mitigation and governance measures through explicit contracts between the university and students. In these contracts, the students are constituted only as targets of the enforcement measures rather than implementers or initiators of the measures. Such contracts can be more or less formalized and documented, but necessarily reinforce the mythology surrounding contractual arrangements as affirming the mutuality of the agreement and the “equal endowment” each party brings to the agreement (Stinchcombe, 2001; Suchman, 2003, p. 111). In the pre-vaccination phase of the pandemic, these contracts took the form of “behavioral contracts” (Lederer & Stolow, 2021) which laid out particular behavioral guidelines pertaining to mask wearing, social distancing, self-testing, self-quarantine, and self-sanitizing. During the post-vaccination phase, vaccination and testing requirements have been added to these contracts.

From the standpoint of Coleman’s theory, the college environment is one where the governance scheme differs from the pure employment settings which are home to symmetric encounters between organizational agents as well as public-facing retail settings which are the locus for asymmetric encounters between organizational agents and natural persons. In the college setting, a single corporate actor is responsible for the health and safety of students (or clients), who are treated as natural persons. However, unlike the natural persons in household settings, students are contractually bound by their association with the corporate body—the university—which takes responsibility for ensuring the collective safety of all students along with faculty and staff. Where the students are concerned, the university approaches them as natural persons rather than agents. But, unlike customers, the students are bound by voluntary contractual agreements which constitute them as governance targets and, to a much lesser extent, as implementers of the measures.^14^ As consenting adults, these students are obliged to agree to the terms of the contract if they wish to continue their education at the university, which serves as the ultimate enforcing actor.

The ultimate authority to enforce the mitigation measures rests with the corporate body, namely the university itself. Violation of the contract between each student and the university can precipitate penalties up to and including termination of enrollment or “revocation” of students’ ties to the university.^15^ Since the onset of the post-vaccination phase, some universities have enforced vaccination requirements through denying unvaccinated students the ability to register for classes or continue as a student at the university.

Under the contractual governance scheme of the university, students are obligated to comply with the measures, but have no responsibility for policing others’ compliance. While students can report each other for violations, such reporting is entirely voluntary. In some universities, however, during the mandate stage of the pandemic, students have been encouraged to report each other for violations,^16^ though they are neither required to do so nor are penalized for not reporting incidents. Indeed, universities have shown little appetite for putting students in the position of “snitching” (Pershing, 2003) on their fellow students and forcing them to weigh their allegiance to their family and friends against their loyalty to the public health of the community.

In this contractual governance scheme, therefore, natural persons who are tied to the corporate entity are treated exclusively as targets of the governance measures, and do not occupy multiple governance roles in the same way as employees in a public-facing establishment. We can say that this governance scheme is thus less agent-centered than the scheme applying to employee–customer or workplace settings, since the students are not obligated to monitor each other for violations of the measures.

## Conclusion: The Evolution of Risk Governance Schemes

As the survey of these settings demonstrates, governance schemes vary in terms of both complexity—in relation to the types of social actors they involve—and in terms of clarity—in relation to how well these actors are specified. While some governance schemes may be classified as simple governance schemes involving natural persons and therefore the domain of simple social relations, other governance schemes necessarily involve constructed social actors and therefore implicate higher-level social entities. In the simplest governance scheme, natural persons formulate governance and mitigation measures and, as targets of such measures, monitor their own compliance and that of their fellow natural persons. In more complex governance schemes, for example those prevailing in universities, natural persons still serve as the targets of governance measures, but they also are called upon to voluntarily monitor the compliance of other natural persons with an analogous tie to the organization. This type of governance scheme is rooted in contractual ties between natural persons and a corporate actor, but implicates these natural persons as both targets and implementers. Moving up the complexity scale, we find pure employment environments comprising agents and principals. We also find employment environments where organizational agents deal with customers *qua* natural persons. In such settings, the operative governance scheme is one where agents of the organization are both contractually bound to honor governance measures themselves—as targets—but also serve as implementers of the measures vis-à-vis other agents and potentially customers as well on behalf of the corporate entity. In this type of setting, the organizational principals bear legal liability, but only for violations attributable to themselves or the agents who are tied specifically to them. Customers may be the targets of governance and mitigation measures, but they cannot be called upon to implement them with respect to other customers or agents of other corporate entities. Just as these risk governance schemes vary in terms of complexity, they also differ in terms of the degree to which the focal social actors align with Coleman’s taxonomy of social actors. Some government-initiated mandates sidestep the distinctions between employees/agents and natural persons which figured prominently in some other types of governance measures such as employer mandates. Other governance measures applying to households and private homes do not offer clear indications as to whether the targets or implementers of the directives ought to be considered either purely natural persons or citizen-sovereign individuals acting in their capacity as members of a joint political collectivity. Thus, ambiguity is evident along two distinct analytical dimensions: the types of social actor addressed in the directive and the particular governance roles occupied by the social actor.

Ranging across a variety of social contexts and settings, the study has pinpointed the ways in which governance roles intersect with different forms of social actorhood. The case study exposition has shown that Coleman’s theory of social actorhood remains theoretically and conceptually more valuable than ever, given the challenges of risk mitigation and governance during the COVID-19 pandemic. It is difficult if not impossible to comprehend the vagaries of exposure risk governance across diverse social environments unless we grapple with the foundational insights of Coleman’s approach to social actorhood. When the notion of governance roles is brought together with the tier identity framework proposed in Coleman’s theory of social actorhood, it is immediately evident that risk governance schemes vary in theoretically important ways.

## Notes

1. This chain or web structure resembles the structure of the interaction chains elucidated by Randall Collins (Collins, 2020) as well as the functional interdependency chains examined in the work of Norbert Elias (Elias, 1978).

2. The US federal agency OSHA promulgated a mandate in November 2021 which applied specifically to places of employment with more than one hundred individual employees. This mandate was blocked by the US Supreme Court as of January 13, 2022. Interestingly, the decision turned on whether COVID should be considered a risk attached to work or employment, as opposed to a free-floating risk which could arise outside the employment context (595 U.S.____ (January 13th, 2022).

3. Empirical research since the beginning of the pandemic suggests that business compliance with government guidelines and mandates is far from universal in many parts of the US, even states which are ostensibly “pro-mandate.” In the state of California, for example, over 100 employers, ranging from hospitals, to farms, to manufacturing workplaces have received citations and fines for COVID-related safety violations since the inception of the pandemic. A recent report on low-wage workers in California based on surveys has found that a large proportion of employers in sectors such as restaurants, landscaping, and domestic services have been routinely flouting guidelines regarding minimizing disease exposure risk for their employees (ALC Report, 2021). Furthermore, government agencies may not learn of violations because of inadequate surveillance and reporting. Instances of actual retaliation against employees who report their employers to government authorities and anxiety about potential retaliation are commonplace in such workplaces (ALC Report, 2021). Finally, in order for a business organization to be held legally liable for sickening a customer, the firm has to be found in violation of the applicable local health guidelines and an explicit causal link must be established between the customer’s illness and the actions of the employee(s) acting on behalf of the organization. The more diffuse and widespread the disease, the more difficult it becomes for the customer to demonstrate this causal link to the satisfaction of legal authorities. In the case of COVID-19, a customer’s claim of infection at a place of business would necessitate excluding the possibility of infection at other sites, a challenge to prove unless the customer’s only social interactions take place in this one location.

4.
https://www.dallascounty.org/departments/dchhs/2019-novel-coronavirus/businesses-and-employers.php

5. See https://www.law.cornell.edu/uscode/text/42/12181).

6. https://www.latimes.com/opinion/story/2021-12-22/editorial-its-time-for-a-covid-vaccination-mandate-on-domestic-flights.

7. San Francisco Chronicle July 24^th^, 2021. Even in some states with relatively lax enforcement policies like Texas, it has happened that businesses have been shut down and business owners have been arrested for noncompliance. See https://www.texastribune.org/2020/05/14/texas-coronavirus-enforcement/.

8. Even in states like Florida where local health authorities are not permitted to sanction private businesses for not requiring proof of vaccination status for customers, private businesses are nonetheless allowed to require attestations of vaccination.

9. See https://www.sfdph.org/dph/alerts/coronavirus-faq.asp

10. See https://dallas.eater.com/2021/8/11/22620163/dallas-county-mask-mandate-judge-clay-jenkins-masks-required-indoors

11. See https://corporate.homedepot.com/newsroom/mask-policy-update-associates-urged-to-vaccinate

12.
http://www.leg.state.fl.us/Statutes/index.cfm?App_mode=Display_Statute&Search_String=&URL=0300-0399/0381/Sections/0381.00316.html

13. There is an interesting overlap between these types of social settings and what epidemiologists call “congregate settings” where individuals engage in repeated interactions in close quarters and continuously occupy the same physical space and breathe the same air.

14. Under US law, the students’ relationship to the university is regarded as a legally enforceable contractual relationship between a natural person and a corporate body (Buchter, 1973). However, the university faculty could be considered—under certain conditions—“principals” of the university’s corporate body, although for strictly legal purposes universities are “owned” by their Boards of Trustees.

15. For example, https://www.umass.edu/coronavirus/interim-pandemic-policy and https://policy.brown.edu/policy/covid-19.

16. See https://www.bu.edu/dos/2020/08/26/violations-repercussions-covid-19/
